# A survey of factors influencing career preference in new-entrant and exiting medical students from four UK medical schools

**DOI:** 10.1186/1472-6920-14-151

**Published:** 2014-07-23

**Authors:** Jennifer A Cleland, Peter W Johnston, Micheal Anthony, Nadir Khan, Neil W Scott

**Affiliations:** 1 Division of Medical and Dental Education, University of Aberdeen, Polwarth Building, West Wing, Foresterhill, Aberdeen AB25 2AZ, UK; 2 NHS Education for Scotland, North Deanery, Forest Grove House, Foresterhill, Aberdeen, UK; 3 Medical Statistics Team, Division of Applied Health Sciences, University of Aberdeen, Foresterhill, Aberdeen, UK

## Abstract

**Background:**

Workforce planning is a central issue for service provision and has consequences for medical education. Much work has been examined the career intentions, career preferences and career destinations of UK medical graduates but there is little published about medical students career intentions. How soon do medical students formulate careers intentions? How much do these intentions and preferences change during medical school? If they do change, what are the determining factors? Our aim was to compare medical students’ career preferences upon entry into and exit from undergraduate medical degree programmes.

**Methods:**

This was a cross-sectional questionnaire survey. Two cohorts [2009–10, 2010–11] of first and final year medical students at the four Scottish graduating medical schools took part in career preference questionnaire surveys. Questions were asked about demographic factors, career preferences and influencing factors.

**Results:**

The response rate was 80.9% [2682/3285]. Significant differences were found across the four schools, most obviously in terms of student origin [Scotland, rest of UK or overseas], age group, and specialty preferences in Year 1 and Year 5. Year 1 and Year 5 students’ specialty preferences also differed within each school and, while there were some common patterns, each medical school had a different profile of students’ career preferences on exit. When the analysis was adjusted for demographic and job-related preferences, specialty preferences differed by gender, and wish for work-life balance and intellectual satisfaction.

**Conclusions:**

This is the first multi-centre study exploring students’ career preferences and preference influences upon entry into and exit from undergraduate medical degree programmes. We found various factors influenced career preference, confirming prior findings. What this study adds is that, while acknowledging student intake differs by medical school, medical school itself seems to influence career preference. Comparisons across medical school populations must therefore control for differences in input [the students] as well as context and process [the medical school] when looking at output [e.g., performance]. A robust, longitudinal study is required to explore how medical students’ career preferences change as they progress through medical school and training to understand the influence of the learning environment on training choice and outcomes.

## Background

Workforce planning is a central issue for service provision and has consequences for medical education, particularly when considering issues such as increasing numbers of women in the medical workforce [1], the increasing popularity of part-time training and working [2], over-supply of doctors in some specialities and localities e.g., [3], dependency on international medical graduates in others [4] and, conversely, overproduction of medical graduates and of doctors qualified for consultant posts, in the UK at least [5]. Additionally, in many countries, the cost of employing doctors have escalated over recent years, in part as a result of working time directives which have limited the hours doctors are available for service [6,7].

The factors which influence career decisions are multiple, ranging from individuals’ characteristics [8-10], to the perceived benefits and attractiveness of particular specialties [11-13], to factors associated with medical school curricula, such as experience of the chosen specialty [13-16]. Recently, studies have suggested that quality of life has become a major determinant in why doctors chose a particular specialty [12,17-20]; this has been found to be more influential than more traditional specialty-linked motivators, such as remuneration [12,17,18]. It is also clear that demographic factors such as gender influence medical career preference [8,18,20,21]. A number of studies, most notably those from the UK Medical Careers Research Group [http://www.uhce.ox.ac.uk/ukmcrg], have explored the complex interplay between these myriad factors relating to graduating doctors’ career choices and prior to this Parkhouse and colleagues carried out much UK work on this topic e.g., [22,23]. However, there is little published about how soon medical students formulate careers intentions or how much career preferences change during medical school - and if they do, what the determining factors are.

Our earlier work identified that Year 1 medical students have definite preferences for and against some specialties, which were probably formed prior to entering medical school , see also [20,24]. While there is evidence, mostly from the US [25-29], that the career preferences of students entering medicine are reasonably firm, findings from graduate-entry medical students studying non-integrated degree programmes are unlikely to be directly applicable to other medical education settings such as the UK, where most students enter medicine as undergraduates aged 17–20 years. Those few studies of career preferences within the UK context have tended to focus on general practice/primary care intentions [30,31], rather than looking across the spectrum of specialties. Moreover, rather than investigating career intentions at the time of active career decisions, many of the studies on specialty preference have carried out pre- and post- clerkship/rotation surveys [32-36] which provide insight into local student experiences but arguably do not progress general understanding of careers decision making.

While there is already evidence of differences between UK medical schools in the career choices and performance of graduates [37,38], the reasons for these differences are unclear in a system where all medical schools must demonstrate compliance with the same published guidance and outcome standards [GMC: http://www.gmc-uk.org/education/undergraduate.asp]. Is the student intake different? Is it the time at medical school which influences students’ career choices? What are the influencing factors at undergraduate level?

There is some indirect evidence for student intake differing: in the UK, medical student demographics vary between medical schools [20,39]; applicants to medicine give different medical schools preferential ranking [40]; UK medical schools use a variety of selection methods [for example, at the time of data collection, only a few schools had adopted newer methods of selection such as mini-multiple interviews MMIs: [41] and use the same selection methods in different ways e.g., [42]. Conversely, are differences in graduate careers a result of the nature of teaching and learning experiences in different specialties and different learning environments? There is certainly evidence of complex influences and pressures at medical school which may influence careers decision making [43,44]. As per Bourdieu and Brosnan [45,46], however, is there an interaction between the two: have different UK medical schools developed their own “habitus” [culture] in order to attract the type of capital [e.g., students] which will fulfil their [the university’s] needs and desires [for example, to be recognised at the best school in the country for producing surgeons]? Exploring this area requires meaningful comparisons both between and within schools in terms of comparing their intake [new-entrants] and their output [exiting medical students].

Why is it important to know about how career preferences in medical students change across the duration of undergraduate degree programmes, or about the relationship between career preferences, and changes in these preferences, and medical school? Health services need a supply of medical graduates willing to train in all specialties, in the right proportions and, crucially, in the right places, to meet healthcare needs. This may be more amenable to modification at the stage of selection to and progression through medical school than after graduation given that the landscape of medical careers, particularly in the UK, is changing rapidly. The changes in medical careers structure and progression resulting from the UK’s “Modernising Medical Careers” [MMC] initiative [47] in 2005 [Foundation implementation] and 2007 [specialty implementation], means junior doctors now have to make a definitive choice about a career pathway much sooner than many did in the past, and indeed the first major careers decision takes place early on in the final year of medical school [see later for further explanation of the re-organisation of UK medical training]. This is coupled with less flexibility to change specialty in the early years of training now compared to prior to MMC. This requirement for earlier career choices means that undergraduate experience is likely to be more influential on careers choice than seemed to be the case formerly [48-50].

It is timely to review curricula in undergraduate and postgraduate contexts to understand how best to best support new doctors in planning their careers and developing the flexible behaviours necessary to deal with evolving health care configurations and careers structures. Careers management approaches and support, in terms of timing and content, might need to be reconsidered.

Thus, the aim of this study was to explore students’ career preferences and the variables influencing these preferences upon entry into and exit from undergraduate medical degree programmes in Scotland using a cross-sectional questionnaire survey.

## Methods

### Setting and background population

Students were surveyed at the four Scottish graduating medical schools [Aberdeen, Dundee, Edinburgh and Glasgow] which together have approximately 850 students in each year of study.

Note that all medical programmes reported in this study are five-year undergraduate degree programmes, as is still the norm in the UK. [At the time of writing this paper, there were 16 graduate entry programmes [GEPs] and 31 undergraduate medical programmes available in the UK, with undergraduate programmes producing approximately 90% of medical graduates].

In the UK, since 2008 [47], the next step after graduating from medical school is Foundation training. These two-year Foundation Programmes [FP] of successive four month placements in a variety of specialties and healthcare settings aim to ensure that recently graduated doctors acquire a broad generic spectrum of clinical knowledge and skills to specified standards of competency, equipping them to practise safely and to a high quality whilst being ready to embark on later Core training [CT] or Specialty training [ST] in hospital medicine or general practice. Acute care is central to all FPs. However, there is an opportunity to vary elements, e.g., some programmes include general practice, psychiatry, laboratory medicine, whilst, others have a focus on academic medicine.

Medical students apply for the FP via a national [UK-wide] allocation system, where they rank their preferences for specific programmes. This process takes place in the first term of final year of medical school. Anecdotal evidence suggests that the experience obtained in FPs [the clinical content] influences career decision making and likelihood of success on application to CT or ST. This places more importance on career decision making in medical school and training experiences immediately post-medical school.

### Participants

All Year 1 and Year 5 medical students at the four Scottish graduating medical schools in 2009–10 and 2010–11.

### Instrument

The development of the questionnaire is described in more detail in an earlier paper [20]. Questions covered the following topics; schools applied to, whether students were at their first choice medical school, specialty preferences [identified by asking students to list their top three choices of medical career which were then broadly categorized e.g., any surgical specialty was categorized under “surgery” to facilitate analysis] and career plans. Students were also asked to rate the importance of the following list of factors influencing careers choice using a four-point Likert scale; intellectual satisfaction, work/life balance, own aptitude/skills, potential earnings, amount of patient contact, continuity of patient contact, career prospects, spouse/partner’s career, location and transport links. No definition of these factors was provided rather their interpretation was left to the individual. Demographic data included; gender, age, ethnicity, socio-economic background and country of birth.

### Data collection

Year 1 medical students at the four Scottish medical schools in 2009–10 and 2010–11 were invited to complete a survey on career preferences within two months of commencing their studies. Year 5 students in 2009–10 and 2010–11 were also invited to complete the survey approximately one month before completing their medical programme. Paper questionnaires were distributed at pre-arranged sessions at each school. Students were emailed details of the study one week before the data collection session. The purpose of the study was explained and a written information sheet provided both by email, in advance, and in the data collection session. Informed consent was implied by questionnaire completion, deemed acceptable by the research ethics committee [see later]. Students were asked to complete the questionnaire within this session, and return it before leaving. No reminders were sent to students who did not attend the session or complete the questionnaire.

### Data analysis

No statistically significant differences were identified across year groups so the data from 2009–10 and 2010–11 were merged for analysis. Age was categorised as 17–21, and 22 and over [39]. Socio-economic class [SEC] was defined using the UK Standard Occupational Classification, which assigns socio-economic status based on an applicant's parental occupation [or the occupation of the person contributing the highest income to the household if the applicant is aged 21 years or over].

Quantitative data were analysed using SPSS, Version 20.0.

Chi-Squared tests [bi-variate analysis] were used to identify statistically significant differences between medical schools and demographic factors [gender, age group, country of birth, ethnic group, SEC, medical school, factors influencing career choice and specialty choice]. Separate analyses were conducted for Year 1 and Year 5 students.

Logistic regression models were then developed to determine which factors were associated with each specialty being a top three choice. The independent variables to be included in the logistic regression analysis were informed by previous literature and chi Square tests which produced statistically significant results. Thus, the following were included in each model: gender, socio-economic class, age group, ethnicity, country of birth, medical school, and whether or not the following future job-related preferences were extremely important [intellectual satisfaction, work/life balance, own aptitude/skills, potential earnings and geographic location]. The logistic regression analysis was an enter model not a stepwise model. Tables present the data from separate models predicting, for example, the importance of career-related factors in specialty preference.

For each medical school, separate chi-squared tests were conducted to determine whether the proportion of respondents selecting each specialty as a top three choice differed by year group.

Due to the large number of statistical comparisons, a reduced level of significance was applied and statistical significance accepted at p < 0.01. Data is reported as statistically significant at p < 0.01 unless otherwise stated [51].

Due to space considerations and the number of different models in the analysis, only statistically significant results are reported. Full tables are available as an Additional file 1. The variables included in each model are presented in the accompanying tables.

### Ethical review

Ethical approval for this study was granted by the College of Life Sciences and Medicine Ethics Review Board [CERB], University of Aberdeen, and approved by the Chairman of the University of Edinburgh Ethics Committee. These permissions were accepted as proof of review by the other medical schools.

## Results

### Response rate

In Year 1, the overall response rate was 80.4% [1332 of 1657 potential respondents]. Return rates differed statistically significantly by school [x^2^ = 124.4, d.f. = 3, p ≤ 0.001]: Aberdeen [94.8%] and Glasgow [87.1%] gave higher response rates than Edinburgh [72.1] and Dundee [66.1%].

Year 5 response rate was 81.4% [1325 out of 1628 potential respondents]. Return rates differed statistically significantly by school [x^2^ = 118.2, d.f. = 3, p ≤ 0.001]: Aberdeen [97.7%] and Glasgow [87.7%] gave higher response rates than Edinburgh [71.3%] and Dundee [68.7%].

Thus, the overall response rate was 80.9%.

### Demographic factors

Table 1 outlines respondents’ demographics, presented by medical school and Year group. 59.0% of Year 1 students were female compared to 67.1% of Year 5 students. Most students had commenced medical school between the age of 17–21 years [87.7% of Year 1 students; 86.8% of Year 5 students]. Approximately half of the students in each year group were Scottish [48.8% of Year 1 students; 46.1% of Year 5 students]. Most students were White [81.7% of Year 1 students; 82.8% of Year 5 students]. Most students were from socio-economic class [SEC] groups I and II [managerial or professional] [88.2% of Year 1 students; 85.8% of Year 5 students].

**Table 1:** Respondent demographics by medical school and year group

**Year 1**	**Respondents**					
	**Aberdeen [n = 347]**	**Dundee [n = 213]**	**Edinburgh [n = 345]**	**Glasgow [n = 424]**	**Total [n = 1329]**	**Statistics**
Gender, *n* [%]						x^2^ = 7.123
Female	208 [59.9]	136 [63.8]	211 [61.2]	227 [54.0]	782 [59.0]	d.f. = 3
Male	139 [40.1]	77 [36.2]	134 [38.8]	193 [46.0]	543 [41.0]	*p* = 0.068
Age group, years, *n* [%]						x^2^ = 23.786
≤21	295 [85.0]	190 [89.2]	326 [94.5]	355 [83.7]	1166 [87.7]	d.f. = 3
>21	52 [15.0]	23 [10.8]	19 [5.5]	69 [16.3]	163 [12.3]	*p* ≤ 0.001
Country of birth, *n* [%]						x^2^ = 26.369
Scotland	183 [52.9]	109 [51.7]	174 [50.7]	176 [42.3]	642 [48.8]	d.f. = 6
Rest of UK	89 [25.7]	59 [28.0]	108 [31.5]	174 [41.8]	430 [32.7]	*p* ≤ 0.001
Other	74 [21.4]	43 [20.4]	61 [17.8]	66 [15.9]	244 [18.5]	
Ethnic group, *n* [%]						x^2^ = 14.956
White	293 [84.4]	179 [85.2]	259 [74.9]	347 [83.2]	1078 [81.7]	d.f. = 3
Other	54 [15.6]	31 [14.8]	87 [25.1]	70 [16.8]	242 [18.3]	*p* = 0.002
Socio-economic status, class, *n* [%]						x^2^ = 7.578
I and II	302 [87.5]	174 [83.7]	316 [91.3]	369 [88.5]	1161 [88.2]	d.f. = 3
Other	43 [12.5]	34 [16.3]	30 [8.7]	48 [11.5]	155 [11.8]	*p* = 0.056
**Year 5**	**Aberdeen [n = 285]**	**Dundee [n = 257]**	**Edinburgh [n = 244]**	**Glasgow [n = 215]**	**Total [n = 1001]**	**Statistics**
Gender, *n* [%]						x^2^ = 13.056
Female	170 [59.6]	171 [66.5]	172 [70.5]	159 [74.0]	672 [67.1]	d.f. = 3
Male	115 [40.4]	86 [33.5]	72 [29.5]	56 [26.0]	329 [32.9]	*p* = 0.005
Age group, years, *n* [%]						x^2^ = 23.838
≤21	242 [86.4]	209 [81.3]	230 [95.4]	179 [84.0]	860 [86.8]	d.f. = 3
>21	38 [13.6]	48 [18.7]	11 [4.6]	34 [16.0]	131 [13.2]	*p* ≤ 0.001
Country of birth, *n* [%]						x^2^ = 54.849
Scotland	125 [44.2]	129 [50.0]	80 [33.3]	125 [58.4]	459 [46.1]	d.f. = 6
Rest of UK	83 [29.3]	93 [36.0]	121 [50.4]	63 [29.4]	360 [36.2]	*p* ≤ 0.001
Other	75 [26.5]	36 [14.0]	39 [16.2]	26 [12.1]	176 [17.7]	
Ethnic group, *n* [%]						x^2^ = 10.949
White	225 [80.1]	214 [84.6]	192 [78.7]	189 [89.2]	820 [82.8]	d.f. = 3
Other	56 [19.9]	39 [15.4]	52 [21.3]	23 [10.8]	170 [17.2]	*p* = 0.012
Socio-economic status, class*, n* [%]						x^2^ = 6.620
I and II	226 [83.1]	219 [87.6]	215 [89.6]	174 [82.9]	834 [85.8]	d.f. = 3
Other	46 [16.9]	31 [12.4]	25 [10.4]	36 [17.1]	138 [14.2]	*p* = 0.085

For Year 1, statistically significant differences were found between medical schools for country of birth [Ҳ^2^ = 26.369, d.f. = 6, *p* ≤ 0.001], age group [Ҳ^2^ = 23.786, d.f. = 3, *p* ≤ 0.001] and ethnicity [Ҳ^2^ = 14.956, d.f. = 3, *p* = 0.002]. Aberdeen Year 1 students were most likely to be Scottish-born [52.9%] and Glasgow students were least likely [42.3%]. Glasgow had the highest proportion of older students [16.3%] [aged 22 or over at time of entry to medical school], Edinburgh the least [5.5%]. Edinburgh had the highest proportion of ethnic minority [25.1%] students, Dundee the least [14.8%].

For Year 5, statistically significant differences were found between medical schools for country of birth [Ҳ^2^ = 54.849, d.f. = 6, *p* ≤ 0.001], age group [Ҳ^2^ = 23.838, d.f. = 3, *p* ≤ 0.001] and gender [Ҳ^2^ = 13.056, d.f. = 3, *p* = 0.005]. Year 5 Glasgow students were most likely to be Scottish-born [58.4%] and Edinburgh students were least likely [33.3%]. Dundee had the highest proportion of older students [18.7%] [aged 22 or over at time of entry to medical school] in Year 5, Edinburgh the least [4.6%]. Edinburgh had the highest proportion of ethnic minority [21.3%] students, Glasgow the least [10.8%] in Year 5. The gender difference was due to particularly high numbers of female Year 5 students at two universities [Glasgow [74%] and Edinburgh [70.5%].

### Career preference

Students were asked to indicate their top three specialty choices. These are presented for each of year 1 and year 5 students [Table 2].

**Table 2:** Year 1 and 5 speciality choices, presented in descending popularity left to right

	**Year 1**							
**Specialty**	**Medicine**	**GP**	**Surgery**	**Paediatrics**	**Emergency medicine**	**Obstetrics and gynae**	**Anaesthetics**	**Diagnostics**
Top 3 count *n*	822	603	483	430	334	127	111	75
%	61.7	45.3	36.3	32.3	25.1	9.5	8.3	5.6
	**Year 5**							
**Specialty**	**Medicine**	**GP**	**Emergency medicine**	**Anaesthetics**	**Surgery**	**Paediatrics**	**Obstetrics and gynae**	**Diagnostics**
Top 3 count *n*	603	559	416	334	268	263	222	80
%	59.2	55.3	41.1	33.0	26.5	26.0	22.0	7.9

Numbers refer to those choosing the specialty as top three choice (%).

Medicine [61.7%], general practice [45.3%] and surgery [36.3%] were most frequently mentioned as top choices by Year 1 students. Medicine [59.6%], general practice [55.3%] and emergency medicine [41.1%] were most frequently mentioned as top choices by Year 5 students.

Tables 3 and 4 show the results of the logistic regression models predicting whether or not a given speciality was included as a top three choice. Only statistically significant (p < 0.01) predictors have been presented. Aberdeen first years were more likely to select general practice as a top three choice compared to Dundee or Glasgow students. On the other hand, Year 1 students in Dundee and Glasgow were more likely to express a preference for emergency medicine than Aberdeen students. Year 1 Dundee students were also more likely to select surgery as a top three choice than Aberdeen students. The patterns were somewhat different in Year 5 students. In Year 5, Aberdeen students were more likely to select general practice as a top three choice than Edinburgh students but less likely to select Anaesthesia compared to Dundee final year students.

**Table 3:** Results of eight logistic regression models predicting whether a specialty is top three choice for Year 1 students (only factors with p < 0.01 shown)

**Dependent variable**	**Independent variable**	**B**	**OR [95% CI]**	**p-value**
**Anaesthesia**				
	Glasgow [ref. Aberdeen]	−1.105	0.331 [0.185-0.592]	p < 0.001
**Emergency medicine**				
	Dundee [ref. Aberdeen]	0.586	1.797 [1.192-2.710]	p = 0.005
	Glasgow [ref. Aberdeen]	0.684	1.983 [1.399-2.810]	p ≤ 0.001
	Other ethnicity [ref. White]	-.642	0.526 [0.355-0.780]	p = 0.001
**General practice**				
	Dundee [ref. Aberdeen]	−0.647	0.524 [0.361-0.759]	p = 0.001
	Glasgow [ref. Aberdeen]	−0.436	0.647 [0.476-0.879]	p = 0.005
	Male gender [ref. Female]	−0.555	0.574 [0.452-0.729]	p ≤ 0.001
	Other ethnicity [ref. White]	−0.959	0.383 [0.274-0.536]	p ≤ 0.001
	Intellect not extremely important [ref. Extremely important]	0.786	2.196 [1.601-3.011]	p ≤ 0.001
	Work-life balance not extremely important [ref. Extremely important]	−0.627	0.534 [0.401-0.711]	p ≤ 0.001
**Medical specialties**	Work-life balance not extremely important [ref. Extremely important]	−0.759	0.468 [0.347-0.631]	p ≤ 0.001
**Surgical specialties**				
	Dundee [ref. Aberdeen]	0.494	1.640 [1.140-2.359]	p = 0.008
	Male gender [ref. Female]	0.637	1.891 [1.493-2.394]	p ≤ 0.001
**Obstetrics & gynaecology**	Male gender [ref. Female]	−1.374	0.253 [0.154-0.4160	p ≤ 0.001
**Paediatrics**	Male gender [ref. Female]	−0.871	0.419 [0.323-0.542]	p ≤ 0.001
**Diagnostics**	Edinburgh [ref. Aberdeen]	−0.895	0.409 [0.204-0.817]	p = 0.010

OR = odds ratio; 95% CI = 95% confidence interval.

See Additional file 1 for on-line supplementary versions of Tables 3 and 4, showing odds ratios for all the predictors in each specialty*.*

**Table 4:** Results of eight logistic regression models predicting whether a specialty is top three choice for Year 5 students (only factors with p < 0.01 shown)

**Dependent variable**	**Independent variable**	**B**	**OR [95% CI]**	***p value***
**Anaesthesia**				
	Dundee [ref. Aberdeen]	0.617	1.853 [1.262-2.721]	*p* = 0.002
	Work-life balance not extremely important [ref. Extremely important]	0.421	1.524 [1.111-2.091]	*p* = 0.009
**Emergency medicine**	Male gender [ref. Female]	0.647	1.910 [1.429-2.553]	*p* ≤ 0.001
**General practice**				
	Country of birth other [ref. Scotland]	−0.837	0.433 [0.256-0.732]	*p* = 0.002
	Edinburgh [ref. Aberdeen]	−0.577	0.562 [0.366-0.861]	*p* = 0.008
	Male gender [ref. Female]	−0.531	0.588 [0.426-0.812]	*p* = 0.001
	Intellect not extremely important [ref. Extremely important]	0.970	2.638 [1.885-3.692]	*p* ≤ 0.001
	Work-life balance not extremely important [ref. Extremely important]	−1.531	0.216 [0.154-0.303]	*p* ≤ 0.001
**Medical specialities**	Country of birth other [ref. Scotland]	0.672	1.957 [1.195-3.207]	*p* = 0.008
**Surgical specialities**	Male gender [ref. Female]	1.191	3.291 [2.372-4.568]	*p* ≤ 0.001
	Other ethnicity [ref. White]	0.677	1.968 [1.190-3.255]	*p* = 0.008
	Work-life balance not extremely important [ref. Extremely important]	0.823	2.278 [1.613-3.216]	*p* ≤ 0.001
**Obstetrics & gynaecology**	Male gender [ref. Female]	−1.561	.210 [0.133-0.332]	*p* ≤ 0.001
**Paediatrics**				
	Male gender [ref. Female]	−0.853	0.426 [0.295-0.616]	*p* ≤ 0.001
**Diagnostics**	Edinburgh [ref. Aberdeen]	−1.109	0.330 [0.155-0.703]	*p* = 0.004

odds ratio; 95% CI = 95% confidence interval.

See Additional file 1 for on-line supplementary versions of Tables 3 and 4, showing odds ratios for all the predictors in each specialty*.*

Specialty preferences differed by gender (see Tables 3 and 4). For both Year 1 and Year 5 students, males were more likely than females to select surgery as a top three choice but less likely to select general practice, Obstetrics and Gynaecology or paediatrics. In Year 5, male students were more also likely to select emergency medicine as a top three choice than female students.

Ethnicity and country of birth were also associated with specialty preference but to a lesser extent (see Tables 3 and 4). In Year 1, White students were more likely to select emergency medicine and general practice as top three choices than non-White students. This pattern was not apparent in Year 5 students where the only statistically significant difference between White and non-White students was in a preference for surgery. Country of birth was not associated with any preferences in Year 1 students whereas in Year 5, Scottish students were more likely to select general practice as a top three choice and less likely to select medical specialties than students from the rest of the UK or overseas.

Specialty preferences were also related to work-life balance and intellectual satisfaction (see Tables 3 and 4). In both Year 1 and Year 5, students for whom work-life balance was extremely important but for whom intellectual satisfaction was not extremely important were more likely to select general practice as a top three choice. In Year 5, the desire for work-life balance was also associated with a preference for anaesthesia. In contrast, Year 5 students who did not view work-life balance as extremely important were more likely to select surgery as a top three choice.

## Discussion

### What this paper adds

This is the first multi-centre study exploring undergraduate medical students’ career preferences and influences on these preferences upon entry into and exit from undergraduate medical degree programmes. It is part of a programme of work exploring career aspirations in doctors-to-be following major changes in UK medical career organization and structure.

While there are clear common patterns of more females than males and students coming from high social classes, there are also differences across the four schools, most obviously in terms of student origin, age and specialty preferences on entry to medical school. This supports the hypothesis that the capital [in this case, the student intake] of medical schools is different. However, the data also suggests that medical school itself influences career preference. Year 1 and Year 5 students’ specialty preferences differed within each school and, while there were some common patterns (e.g., interest in Anaesthesia was greater in Year 5 students at every medical school, interest in O&G was greater in Year 5 students in three of the four medical schools), each medical school had a different profile of students’ career preferences on exit. Thus, differences in exiting student career preferences between medical schools are related both to capital [student variables] *and* habitus [variation in medical school education and culture] [45,46].

### Comparison with existing literature

As found in previous studies, many of the students in this survey regarded work-life balance [11,17-20,22-26,48-52] as an important factor in career preference. This is in keeping with the wider literature on “Generation Y” for those born after the early 1980s [53,54]. Generation Y has strong social pressures, with active family roles, and may be motivated by a fulfilled and well-balanced life. While specialties such as the surgery group have traditionally been seen as highly desirable, it seems that the importance of work-life balance to tomorrow’s doctors is now influencing the development of career aspirations for many [but not all] medical students.

However, work-life balance in surgery is much more attainable now given the restrictions in training and working hours [6,7]. It may be that today’s students are basing their perceptions of surgery on the messages from role models [55] from another generation, one raised with different expectations of medical school and practising medicine, and who are likely to be male [compared to the majority of medical students]. Surgery is changing – it may be that different skills are needed for, for example, laparoscopic procedures compared to long operations which require physical strength, and there is much more emphasis on teamwork [56], but it is still being defined in traditional male terms.

General practice is seen as attractive because of its perceived work-life balance but more so for those students who did not rate intellectual stimulation as particularly important in a career. It is acknowledged that this finding is at odds with the statements made by the specialty: intellectual satisfaction is highlighted as a key feature of general practice on many relevant websites [e.g., GP Australia http://www.gpaustralia.org.au/home; The Royal College of General Practitioners in the UK http://www.rcgp.org.uk] but this attribute does not seem to be perceived by undergraduate medical students. This paradoxical finding requires further elucidation.

Traditional gender differences in careers preferences were confirmed [20,22-26,48-52]. Does this mean that a female majority of medical students will result in a scarcity of doctors in certain specialties? As discussed by Riska [2009], this assumption might be found to be related to the previous minority status of female doctors rather than a permanent pattern when the gender balance of the medical profession is that of male minority and different practice styles [e.g., shorter working hours across all specialties] become more prevalent [57]. Furthermore, while the assumption is that women are going to change medicine radically, little research has addressed what male medical students are doing and how their careers are changing [58].

It is worth highlighting that our data also shows no gender differences in those wanting to do the [popular options] of Anaesthetics and Medical Specialties.

Of interest is the finding that ethnicity and country of birth were associated with specialty preference to some extent. Most notably, in fifth year students, those from an ethnic minority [non-White] background were more likely to have a preference for surgery. The raw data shows that this group was mainly of Chinese [21% of non-White Year 1 students and 25% of non-White Year 5 students] or Indian ethnic background [15% of non-White Year 1 students and 16% of non-White Year 5 students]. These ethnic minority groups have patterns of high academic achievement and aspirations emanating from parental and community expectation [59], so it is perhaps unsurprising to see a preference for a “high status” specialty.

### Strengths and weaknesses

An important strength of this study is that we were surveying Year 1 students before they had much experience of medical school and Year 5 students after they had made decisions about their post-graduate Foundation training. The study achieved an excellent response rate. This may have been due to the face-to-face nature of the data collection, which was time-consuming and expensive, but advantageous in terms of ensuring a high response rate. Differences in response rate between schools were due to differences in the timing and internal advertising of, and hence attendance at, the study session. Unfortunately, the ethical approval we obtained for this study did not enable us to request the demographic details of non-respondents from the respective medical schools, or to contact these individuals separately. The proportion of female and White students in the survey is in keeping with the Scottish medical student population and the general Scottish population [60]. We collected data from more than one year group to control for potential cohort effects.

There are different ways of measuring preferences – lists of alternatives or open-ended questions. We opted for the latter as the former would have required an extensive list including specialities which may have been quite unfamiliar to our Year 1 respondents who had little experience of medical training or systems. We asked for top three choices of specialty to gain a greater coverage of students’ preferences rather than just asking “first” choice. The limitation of this approach is that it does result in triple counting of each specialty and we do not know the “weighting” of each choice. Nor can we interpret reasons for very disparate choices – this requires a qualitative, exploratory study. However, no approach to measuring preferences is perfect.

An important weakness – but perhaps also its strength - of this study is that it is context specific: differences in medical admissions and training mean the data are only applicable to the UK system. The nature of the data collected means we cannot say if Year 5 preferences reflect the pattern of applications for particular Foundation Programmes. Nor can we ascertain the causality of differences in student populations across medical schools. Students may apply for certain schools based on their understanding of its reputation in certain subjects, or schools may use different criteria to select between applicants.

While changes in careers preferences between Year 1 and Year 5 students are possibly due, at least to some extent, to experiences in teaching and when on rotation, we cannot identify the specific influences which maintain or change early career preferences.

The strengths and weaknesses of the survey questions are discussed elsewhere [20].

## Conclusions

The findings from this study indicate that differences in graduating student career preferences between medical schools are related both to capital *and* habitus [45]. This is important as it means comparisons across medical school populations must control for differences in input [the students] as well as context and process [the medical school] when looking at output [e.g., performance on national exiting/licensing or postgraduate examinations]. The need to take into account a complexity of variables has long been recognised in the [school] educational effectiveness literature e.g., [61] but is less embedded in medical education.

This study adds further evidence to the debate as to the relative contribution of medical student characteristics [the people we select], undergraduate medical education provision [the educational programmes they follow], the differences in medical school output [the doctors we produce] and their career preferences [the careers they will follow].

A robust, longitudinal study is required to explore how medical students’ career preferences change as they progress through both medical school and training. This would provide more understanding of the influence of variables such as curriculum design and quality of the learning environment on training choice and outcomes [45] at a time where there is increasing recognition of the need for more flexibility within medical careers to meet the changing needs of doctors in training, patients and healthcare providers e.g., [62].

## Competing interests

The authors declare they have no competing interests.

## Authors’ contributions

JC and PJ had the original idea for the study. MA and NK carried out the data collection, cleaning and analysis, and helped prepare the draft manuscript. NS participated in the design of the study and guided the statistical analysis. JC lead on the drafting of the manuscript. All authors read and approved the final manuscript.

## Pre-publication history

The pre-publication history for this paper can be accessed here:

http://www.biomedcentral.com/1472-6920/14/151/prepub

## Supplementary Material

Additional file 1 **Full tables to accompany Tables **3** and **4**.** Cleland A survey of factors influencing career preference Additional tables.doc. Click here for file
