# Supplementary material for: A survey of factors influencing career preference in new-entrant and exiting medical students from four UK medical schools
Source: BMC Med Educ. 2014 Jul 23;14:151. doi: 10.1186/1472-6920-14-151 (PMC4131477; doi:10.1186/1472-6920-14-151)
Supplement: Additional file 1 — Full tables to accompany Tables 3 and 4. Cleland A survey of factors influencing career preference Additional tables.doc. [file 1472-6920-14-151-S1.docx]

**Addtional file 1: On-line supplementary versions of Tables 3 and 4, showing odds ratios for all the predictors in each specialty, presented by specialty and Year group**

**Addtional file 1: Table S1: Logistic regression model predicting whether anaesthesia is top three choice for Year 1 students**

|  | **Coeff** | **df** | **p-value** | **Odds ratio (95% CI)** |
| --- | --- | --- | --- | --- |
| Country of birth |  | 2 | 0.125 |  |
| Rest of UK (Scotland reference) | 0.402 | 1 | 0.084 | 1.50 (0.95, 2.36) |
| Other (Scotland reference) | 0.462 | 1 | 0.102 | 1.59 (0.91, 2.76) |
| Medical school |  | 3 | <0.001 |  |
| Dundee (Aberdeen reference) | -0.670 | 1 | 0.053 | 0.51 (0.26, 1.01) |
| Edinburgh (Aberdeen reference) | 0.083 | 1 | 0.739 | 1.09 (0.67, 1.77) |
| Glasgow (Aberdeen reference) | -1.105 | 1 | <0.001 | 0.33 (0.19, 0.59) |
| Male gender | 0.173 | 1 | 0.402 | 1.19 (0.79, 1.78) |
| Age (22 and over reference) | 0.612 | 1 | 0.038 | 1.84 (1.04, 3.28) |
| SEC (1&2 reference) | -0.953 | 1 | 0.048 | 0.39 (0.15, 0.99) |
| Non-white ethnicity | -0.332 | 1 | 0.254 | 0.72 (0.41, 1.27) |
| Intellect not extremely important | 0.089 | 1 | 0.766 | 1.09 (0.61, 1.97) |
| Aptitude not extremely important | 0.304 | 1 | 0.303 | 1.36 (0.76, 2.42) |
| Earnings not extremely important | -0.075 | 1 | 0.823 | 0.93 (0.48, 1.79) |
| Work-life balance not extremely important | 0.553 | 1 | 0.052 | 1.74 (1.00, 3.04) |
| Location not extremely important | 0.016 | 1 | 0.960 | 1.02 (0.54, 1.91) |
| Constant | -2.944 | 1 | <0.001 |  |

**Addtional file 1: Table S2: Logistic regression model predicting whether emergency medicine is top three choice for Year 1 students**

|  | **Coeff** | **df** | **p-value** | **Odds ratio (95% CI)** |
| --- | --- | --- | --- | --- |
| Country of birth |  | 2 | 0.509 |  |
| Rest of UK (Scotland reference) | -0.142 | 1 | 0.337 | 0.87 (0.65, 1.16) |
| Other (Scotland reference) | -0.176 | 1 | 0.366 | 0.84 (0.57, 1.23) |
| Medical school |  | 3 | <0.001 |  |
| Dundee (Aberdeen reference) | 0.586 | 1 | 0.005 | 1.80 (1.19, 2.71) |
| Edinburgh (Aberdeen reference) | 0.143 | 1 | 0.467 | 1.15 (0.78, 1.70) |
| Glasgow (Aberdeen reference) | 0.684 | 1 | <0.001 | 1.98 (1.40, 2.81) |
| Male gender | 0.243 | 1 | 0.068 | 1.28 (0.98, 1.65) |
| Age (22 and over reference) | -0.221 | 1 | 0.301 | 0.80 (0.53, 1.22) |
| SEC (1&2 reference) | 0.318 | 1 | 0.117 | 1.37 (0.92, 2.04) |
| Non-white ethnicity | -0.642 | 1 | 0.001 | 0.53 (0.34, 0.78) |
| Intellect not extremely important | -0.217 | 1 | 0.205 | 0.81 (0.58, 1.13) |
| Aptitude not extremely important | 0.096 | 1 | 0.574 | 1.10 (0.79, 1.54) |
| Earnings not extremely important | -0.001 | 1 | 0.995 | 1.00 (0.65, 1.53) |
| Work-life balance not extremely important | 0.178 | 1 | 0.270 | 1.19 (0.87, 1.64) |
| Location not extremely important | -0.047 | 1 | 0.802 | 0.95 (0.66, 1.38) |
| Constant | -1.394 | 1 | <0.001 |  |

**Addtional file 1: Table S3: Logistic regression model predicting whether general practice is top three choice for Year 1 students**

|  | **Coeff** | **df** | **p-value** | **Odds ratio (95% CI)** |
| --- | --- | --- | --- | --- |
| Country of birth |  | 2 | 0.435 |  |
| Rest of UK (Scotland reference) | -0.146 | 1 | 0.275 | 0.86 (0.66, 1.12) |
| Other (Scotland reference) | -0.170 | 1 | 0.321 | 0.84 (0.60, 1.18) |
| Medical school |  | 3 | 0.003 |  |
| Dundee (Aberdeen reference) | -0.647 | 1 | 0.001 | 0.52 (0.36, 0.76) |
| Edinburgh (Aberdeen reference) | -0.248 | 1 | 0.131 | 0.78 (0.57, 1.08) |
| Glasgow (Aberdeen reference) | -0.436 | 1 | 0.005 | 0.65 (0.47, 0.88) |
| Male gender | -0.555 | 1 | <0.001 | 0.57 (0.45, 0.73) |
| Age (22 and over reference) | 0.337 | 1 | 0.075 | 1.40 (0.97, 2.03) |
| SEC (1&2 reference) | -0.315 | 1 | 0.109 | 0.73 (0.50, 1.07) |
| Non-white ethnicity | -0.959 | 1 | <0.001 | 0.38 (0.27, 0.54) |
| Intellect not extremely important | 0.786 | 1 | <0.001 | 2.20 (1.60, 3.01) |
| Aptitude not extremely important | -0.070 | 1 | 0.653 | 0.93 (0.69, 1.27) |
| Earnings not extremely important | 0.174 | 1 | 0.379 | 1.19 (0.81, 1.76) |
| Work-life balance not extremely important | -0.627 | 1 | <0.001 | 0.53 (0.40, 0.71) |
| Location not extremely important | 0.095 | 1 | 0.586 | 1.10 (0.78, 1.55) |
| Constant | 0.259 | 1 | 0.352 |  |

**Addtional file 1: Table S4: Logistic regression model predicting whether medical specialties is top three choice for Year 1 students**

|  | **Coeff** | **df** | **p-value** | **Odds ratio (95% CI)** |
| --- | --- | --- | --- | --- |
| Country of birth |  | 2 | 0.976 |  |
| Rest of UK (Scotland reference) | 0.025 | 1 | 0.851 | 1.03 (0.79, 1.33) |
| Other (Scotland reference) | 0.029 | 1 | 0.866 | 1.03 (0.74, 1.44) |
| Medical school |  | 3 | 0.094 |  |
| Dundee (Aberdeen reference) | -0.451 | 1 | 0.016 | 0.64 (0.44, 0.92) |
| Edinburgh (Aberdeen reference) | -0.076 | 1 | 0.648 | 0.93 (0.67, 1.28) |
| Glasgow (Aberdeen reference) | -0.085 | 1 | 0.591 | 0.92 (0.67, 1.25) |
| Male gender | 0.008 | 1 | 0.950 | 1.01 (0.80, 1.28) |
| Age (22 and over reference) | 0.476 | 1 | 0.017 | 1.61 (1.09, 2.38) |
| SEC (1&2 reference) | 0.243 | 1 | 0.233 | 1.28 (0.86, 1.90) |
| Non-white ethnicity | -0.050 | 1 | 0.757 | 0.95 (0.69, 1.31) |
| Intellect not extremely important | -0.154 | 1 | 0.342 | 0.86 (0.62, 1.18) |
| Aptitude not extremely important | -0.274 | 1 | 0.085 | 0.76 (0.56, 1.04) |
| Earnings not extremely important | 0.298 | 1 | 0.129 | 1.35 (0.92, 1.98) |
| Work-life balance not extremely important | -0.759 | 1 | <0.001 | 0.47 (0.35, 0.64) |
| Location not extremely important | 0.111 | 1 | 0.530 | 1.12 (0.79, 1.58) |
| Constant | 1.017 | 1 | <0.001 |  |

**Addtional file 1: Table S5: Logistic regression model predicting whether surgical specialties is top three choice for Year 1 students**

|  | **Coeff** | **df** | **p-value** | **Odds ratio (95% CI)** |
| --- | --- | --- | --- | --- |
| Country of birth |  | 2 | 0.921 |  |
| Rest of UK (Scotland reference) | -0.008 | 1 | 0.955 | 0.99 (0.76, 1.30) |
| Other (Scotland reference) | -0.068 | 1 | 0.690 | 0.94 (0.67, 1.30) |
| Medical school |  | 3 | 0.002 |  |
| Dundee (Aberdeen reference) | 0.494 | 1 | 0.008 | 1.64 (1.14, 2.36) |
| Edinburgh (Aberdeen reference) | -0.230 | 1 | 0.174 | 0.80 (0.57, 1.11) |
| Glasgow (Aberdeen reference) | -0.007 | 1 | 0.964 | 0.99 (0.73, 1.35) |
| Male gender | 0.637 | 1 | <0.001 | 1.89 (1.50, 2.39) |
| Age (22 and over reference) | -0.219 | 1 | 0.254 | 1.89 (1.50, 2.39) |
| SEC (1&2 reference) | 0.149 | 1 | 0.432 | 0.80 (0.55, 1.17) |
| Non-white ethnicity | 0.353 | 1 | 0.026 | 1.42 (1.04, 1.94) |
| Intellect not extremely important | -0.142 | 1 | 0.365 | 0.87 (0.64, 1.18) |
| Aptitude not extremely important | -0.082 | 1 | 0.597 | 0.92 (0.68, 1.25) |
| Earnings not extremely important | -0.232 | 1 | 0.235 | 0.79 (0.54, 1.16) |
| Work-life balance not extremely important | 0.053 | 1 | 0.714 | 1.05 (0.79, 1.40) |
| Location not extremely important | -0.014 | 1 | 0.935 | 0.99 (0.70, 1.38) |
| Constant | -0.571 | 1 | 0.037 |  |

**Addtional file 1: Table S6: Logistic regression model predicting whether obstetrics and gynaecology is top three choice for Year 1 students**

|  | **Coeff** | **df** | **p-value** | **Odds ratio (95% CI)** |
| --- | --- | --- | --- | --- |
| Country of birth |  | 2 | 0.794 |  |
| Rest of UK (Scotland reference) | 0.111 | 1 | 0.613 | 1.12 (0.73, 1.72) |
| Other (Scotland reference) | 0.191 | 1 | 0.480 | 1.21 (0.71, 2.06) |
| Medical school |  | 3 | 0.151 |  |
| Dundee (Aberdeen reference) | -0.573 | 1 | 0.072 | 0.56 (0.30, 1.05) |
| Edinburgh (Aberdeen reference) | -0.113 | 1 | 0.653 | 0.89 (0.55, 1.46) |
| Glasgow (Aberdeen reference) | -0.461 | 1 | 0.075 | 0.63 (0.38, 1.05) |
| Male gender | -1.374 | 1 | <0.001 | 0.25 (0.15, 0.42) |
| Age (22 and over reference) | -0.054 | 1 | 0.864 | 0.95 (0.51, 1.77) |
| SEC (1&2 reference) | -0.029 | 1 | 0.929 | 0.97 (0.52, 1.82) |
| Non-white ethnicity | 0.151 | 1 | 0.553 | 1.16 (0.71, 1.92) |
| Intellect not extremely important | -0.099 | 1 | 0.698 | 0.91 (0.55, 1.49) |
| Aptitude not extremely important | -0.395 | 1 | 0.112 | 0.67 (0.41, 1.10) |
| Earnings not extremely important | 0.171 | 1 | 0.630 | 1.19 (0.59, 2.38) |
| Work-life balance not extremely important | 0.040 | 1 | 0.866 | 1.04 (0.65, 1.66) |
| Location not extremely important | 0.450 | 1 | 0.115 | 1.57 (0.84, 2.92) |
| Constant | -1.928 | 1 | <0.001 |  |

**Addtional file 1: Table S7: Logistic regression model predicting whether paediatrics is top three choice for Year 1 students**

|  | **Coeff** | **df** | **p-value** | **Odds ratio (95% CI)** |
| --- | --- | --- | --- | --- |
| Country of birth |  | 2 | 0.155 |  |
| Rest of UK (Scotland reference) | 0.007 | 1 | 0.958 | 1.01 (0.76, 1.33) |
| Other (Scotland reference) | 0.344 | 1 | 0.047 | 1.41 (1.00, 1.98) |
| Medical school |  | 3 | 0.160 |  |
| Dundee (Aberdeen reference) | -0.324 | 1 | 0.101 | 0.72 (0.49, 1.07) |
| Edinburgh (Aberdeen reference) | -0.075 | 1 | 0.657 | 0.93 (0.67, 1.29) |
| Glasgow (Aberdeen reference) | -0.310 | 1 | 0.059 | 0.73 (0.53, 1.01) |
| Male gender | -0.871 | 1 | <0.001 | 0.42 (0.32, 0.54) |
| Age (22 and over reference) | -0.468 | 1 | 0.028 | 0.63 (0.41, 0.95) |
| SEC (1&2 reference) | 0.077 | 1 | 0.705 | 1.08 (0.72, 1.61) |
| Non-white ethnicity | -0.065 | 1 | 0.702 | 0.94 (0.67, 1.31) |
| Intellect not extremely important | 0.301 | 1 | 0.075 | 1.35 (0.97, 1.88) |
| Aptitude not extremely important | 0.049 | 1 | 0.768 | 1.05 (0.76, 1.45) |
| Earnings not extremely important | 0.089 | 1 | 0.677 | 1.09 (0.72, 1.66) |
| Work-life balance not extremely important | -0.279 | 1 | 0.065 | 0.76 (0.56, 1.02) |
| Location not extremely important | 0.264 | 1 | 0.159 | 1.30 (0.90, 1.88) |
| Constant | -0.646 | 1 | 0.029 |  |

**Addtional file 1: Table S8: Logistic regression model predicting whether diagnostics is top three choice for Year 1 students**

|  | **Coeff** | **df** | **p-value** | **Odds ratio (95% CI)** |
| --- | --- | --- | --- | --- |
| Country of birth |  | 2 | 0.367 |  |
| Rest of UK (Scotland reference) | -0.292 | 1 | 0.330 | 0.75 (0.42, 1.34) |
| Other (Scotland reference) | 0.212 | 1 | 0.500 | 1.24 (0.67, 2.29) |
| Medical school |  | 3 | 0.050 |  |
| Dundee (Aberdeen reference) | -0.601 | 1 | 0.108 | 0.55 (0.26, 1.14) |
| Edinburgh (Aberdeen reference) | -0.895 | 1 | 0.011 | 0.41 (0.20, 0.82) |
| Glasgow (Aberdeen reference) | -0.580 | 1 | 0.057 | 0.56 (0.31, 1.02) |
| Male gender | 0.380 | 1 | 0.123 | 1.46 (0.90, 2.37) |
| Age (22 and over reference) | 0.019 | 1 | 0.957 | 1.02 (0.51, 2.04) |
| SEC (1&2 reference) | 0.443 | 1 | 0.172 | 1.56 (0.82, 2.94) |
| Non-white ethnicity | 0.342 | 1 | 0.260 | 1.41 (0.78, 2.55) |
| Intellect not extremely important | -0.436 | 1 | 0.148 | 0.65 (0.36, 1.17) |
| Aptitude not extremely important | -0.511 | 1 | 0.090 | 0.60 (0.33, 1.08) |
| Earnings not extremely important | -0.113 | 1 | 0.779 | 0.89 (0.41, 1.97) |
| Work-life balance not extremely important | -0.092 | 1 | 0.741 | 0.91 (0.53, 1.57) |
| Location not extremely important | 0.394 | 1 | 0.274 | 1.48 (0.73, 3.01) |
| Constant | -2.171 | 1 | <0.001 |  |

**Addtional file 1: Table S9: Logistic regression model predicting whether anaesthesia is top three choice for Year 5 students**

|  | **Coeff** | **df** | **p-value** | **Odds ratio (95% CI)** |
| --- | --- | --- | --- | --- |
| Country of birth |  | 2 | 0.430 |  |
| Rest of UK (Scotland reference) | 0.150 | 1 | 0.360 | 1.16 (0.84, 1.60) |
| Other (Scotland reference) | 0.292 | 1 | 0.249 | 1.34 (0.82, 2.20) |
| Medical school |  | 3 | <0.001 |  |
| Dundee (Aberdeen reference) | 0.617 | 1 | 0.002 | 1.85 (1.26, 2.72) |
| Edinburgh (Aberdeen reference) | -0.086 | 1 | 0.680 | 0.92 (0.61, 1.38) |
| Glasgow (Aberdeen reference) | -0.189 | 1 | 0.383 | 0.83 (0.54, 1.27) |
| Male gender | 0.338 | 1 | 0.030 | 1.40 (1.03, 1.90) |
| Age (22 and over reference) | 0.197 | 1 | 0.362 | 1.22 (0.80, 1.86) |
| SEC (1&2 reference) | 0.128 | 1 | 0.532 | 1.14 (0.76, 1.70) |
| Non-white ethnicity | -0.063 | 1 | 0.801 | 0.94 (0.58, 1.53) |
| Intellect not extremely important | -0.356 | 1 | 0.032 | 0.70 (0.51, 0.97) |
| Aptitude not extremely important | 0.076 | 1 | 0.662 | 1.08 (0.77, 1.52) |
| Earnings not extremely important | -0.402 | 1 | 0.053 | 0.67 (0.45, 1.01) |
| Work-life balance not extremely important | 0.421 | 1 | 0.009 | 1.52 (1.11, 2.09) |
| Location not extremely important | 0.348 | 1 | 0.025 | 1.42 (1.04, 1.92) |
| Constant | -0.955 | 1 | <0.001 |  |

**Addtional file 1: Table S10: Logistic regression model predicting whether emergency medicine is top three choice for Year 5 students**

|  | **Coeff** | **df** | **p-value** | **Odds ratio (95% CI)** |
| --- | --- | --- | --- | --- |
| Country of birth |  | 2 | 0.031 |  |
| Rest of UK (Scotland reference) | 0.342 | 1 | 0.027 | 1.41 (1.04, 1.91) |
| Other (Scotland reference) | 0.484 | 1 | 0.045 | 1.62 (1.01, 2.60) |
| Medical school |  | 3 | 0.678 |  |
| Dundee (Aberdeen reference) | 0.082 | 1 | 0.664 | 1.09 (0.75, 1.57) |
| Edinburgh (Aberdeen reference) | 0.081 | 1 | 0.676 | 1.08 (0.74, 1.58) |
| Glasgow (Aberdeen reference) | -0.138 | 1 | 0.493 | 0.87 (0.59, 1.29) |
| Male gender | 0.647 | 1 | <0.001 | 1.91 (1.43, 2.55) |
| Age (22 and over reference) | -0.066 | 1 | 0.753 | 0.94 (0.62, 1.42) |
| SEC (1&2 reference) | 0.203 | 1 | 0.296 | 1.23 (0.84, 1.79) |
| Non-white ethnicity | -0.108 | 1 | 0.643 | 0.90 (0.57, 1.42) |
| Intellect not extremely important | -0.093 | 1 | 0.551 | 0.91 (0.67, 1.24) |
| Aptitude not extremely important | -0.163 | 1 | 0.319 | 0.85 (0.62, 1.17) |
| Earnings not extremely important | -0.217 | 1 | 0.276 | 0.81 (0.55, 1.19) |
| Work-life balance not extremely important | 0.340 | 1 | 0.028 | 1.41 (1.04, 1.91) |
| Location not extremely important | 0.016 | 1 | 0.915 | 1.02 (0.76, 1.35) |
| Constant | -0.618 | 1 | 0.015 |  |

**Addtional file 1: Table S11: Logistic regression model predicting whether general practice is top three choice for Year 5 students**

|  | **Coeff** | **df** | **p-value** | **Odds ratio (95% CI)** |
| --- | --- | --- | --- | --- |
| Country of birth |  | 2 | 0.002 |  |
| Rest of UK (Scotland reference) | -0.424 | 1 | 0.013 | 0.66 (0.47, 0.91) |
| Other (Scotland reference) | -0.837 | 1 | 0.002 | 0.43 (0.26, 0.73) |
| Medical school |  | 3 | 0.071 |  |
| Dundee (Aberdeen reference) | -0.315 | 1 | 0.140 | 0.73 (0.48, 1.11) |
| Edinburgh (Aberdeen reference) | -0.577 | 1 | 0.008 | 0.56 (0.37, 0.86) |
| Glasgow (Aberdeen reference) | -0.315 | 1 | 0.162 | 0.73 (0.47, 1.14) |
| Male gender | -0.531 | 1 | 0.001 | 0.59 (0.43, 0.81) |
| Age (22 and over reference) | 0.008 | 1 | 0.973 | 1.01 (0.63, 1.60) |
| SEC (1&2 reference) | 0.025 | 1 | 0.907 | 1.03 (0.67, 1.57) |
| Non-white ethnicity | -0.563 | 1 | 0.029 | 0.57 (0.34, 0.95) |
| Intellect not extremely important | 0.970 | 1 | <0.001 | 2.64 (1.89, 3.69) |
| Aptitude not extremely important | 0.244 | 1 | 0.187 | 1.28 (0.89, 1.83) |
| Earnings not extremely important | 0.528 | 1 | 0.016 | 1.70 (1.10, 2.60) |
| Work-life balance not extremely important | -1.531 | 1 | <0.001 | 0.22 (0.15, 0.30) |
| Location not extremely important | -0.094 | 1 | 0.564 | 0.91 (0.66, 1.25) |
| Constant | 0.696 | 1 | 0.014 |  |

**Addtional file 1: Table S12: Logistic regression model predicting whether medical specialties is top three choice for Year 5 students**

|  | **Coeff** | **df** | **p-value** | **Odds ratio (95% CI)** |
| --- | --- | --- | --- | --- |
| Country of birth |  | 2 | 0.024 |  |
| Rest of UK (Scotland reference) | 0.018 | 1 | 0.906 | 1.02 (0.76, 1.37) |
| Other (Scotland reference) | 0.672 | 1 | 0.008 | 1.96 (1.20, 3.21) |
| Medical school |  | 3 | 0.271 |  |
| Dundee (Aberdeen reference) | -0.062 | 1 | 0.740 | 0.94 (0.65, 1.35) |
| Edinburgh (Aberdeen reference) | 0.216 | 1 | 0.264 | 1.24 (0.85, 1.81) |
| Glasgow (Aberdeen reference) | 0.261 | 1 | 0.191 | 1.30 (0.88, 1.92) |
| Male gender | -0.078 | 1 | 0.602 | 0.93 (0.69, 1.24) |
| Age (22 and over reference) | 0.022 | 1 | 0.918 | 1.02 (0.68, 1.55) |
| SEC (1&2 reference) | 0.081 | 1 | 0.680 | 1.08 (0.74, 1.59) |
| Non-white ethnicity | -0.132 | 1 | 0.578 | 0.88 (0.55, 1.40) |
| Intellect not extremely important | -0.105 | 1 | 0.497 | 0.90 (0.67, 1.22) |
| Aptitude not extremely important | 0.069 | 1 | 0.667 | 1.07 (0.78, 1.47) |
| Earnings not extremely important | 0.124 | 1 | 0.537 | 1.13 (0.77, 1.68) |
| Work-life balance not extremely important | -0.238 | 1 | 0.127 | 0.79 (0.58, 1.07) |
| Location not extremely important | 0.053 | 1 | 0.716 | 1.05 (0.79, 1.40) |
| Constant | 0.224 | 1 | 0.377 |  |

**Addtional file 1: Table S13: Logistic regression model predicting whether surgical specialties is top three choice for Year 5 students**

|  | **Coeff** | **df** | **p-value** | **Odds ratio (95% CI)** |
| --- | --- | --- | --- | --- |
| Country of birth |  | 2 | 0.486 |  |
| Rest of UK (Scotland reference) | 0.049 | 1 | 0.792 | 1.05 (0.73, 1.51) |
| Other (Scotland reference) | 0.320 | 1 | 0.232 | 1.38 (0.82, 2.33) |
| Medical school |  | 3 | 0.464 |  |
| Dundee (Aberdeen reference) | -0.108 | 1 | 0.622 | 0.90 (0.58, 1.38) |
| Edinburgh (Aberdeen reference) | -0.358 | 1 | 0.117 | 0.70 (0.45, 1.09) |
| Glasgow (Aberdeen reference) | -0.181 | 1 | 0.440 | 0.83 (0.53, 1.32) |
| Male gender | 1.191 | 1 | <0.001 | 3.29 (2.37, 4.57) |
| Age (22 and over reference) | -0.001 | 1 | 0.998 | 1.00 (0.62, 1.60) |
| SEC (1&2 reference) | -0.142 | 1 | 0.545 | 0.87 (0.55, 1.37) |
| Non-white ethnicity | 0.677 | 1 | 0.008 | 1.97 (1.19, 3.26) |
| Intellect not extremely important | -0.330 | 1 | 0.072 | 0.72 (0.50, 1.03) |
| Aptitude not extremely important | -0.408 | 1 | 0.039 | 0.67 (0.45, 0.98) |
| Earnings not extremely important | -0.287 | 1 | 0.200 | 0.75 (0.48, 1.16) |
| Work-life balance not extremely important | 0.823 | 1 | <0.001 | 2.28 (1.61, 3.22) |
| Location not extremely important | -0.046 | 1 | 0.793 | 0.96 (0.68, 1.34) |
| Constant | -1.289 | 1 | <0.001 |  |

**Addtional file 1: Table S14: Logistic regression model predicting whether obstetrics and gynaecology is top three choice for Year 5 students**

|  | **Coeff** | **df** | **p-value** | **Odds ratio (95% CI)** |
| --- | --- | --- | --- | --- |
| Country of birth |  | 2 | 0.626 |  |
| Rest of UK (Scotland reference) | -0.165 | 1 | 0.376 | 0.85 (0.59, 1.22) |
| Other (Scotland reference) | -0.183 | 1 | 0.541 | 0.83 (0.46, 1.50) |
| Medical school |  | 3 | 0.047 |  |
| Dundee (Aberdeen reference) | 0.054 | 1 | 0.822 | 1.06 (0.66, 1.69) |
| Edinburgh (Aberdeen reference) | -0.007 | 1 | 0.976 | 0.99 (0.62, 1.60) |
| Glasgow (Aberdeen reference) | 0.547 | 1 | 0.019 | 1.73 (1.09, 2.73) |
| Male gender | -1.561 | 1 | <0.001 | 0.21 (0.13, 0.33) |
| Age (22 and over reference) | -0.158 | 1 | 0.554 | 0.85 (0.51, 1.44) |
| SEC (1&2 reference) | -0.124 | 1 | 0.612 | 0.88 (0.55, 1.43) |
| Non-white ethnicity | -0.002 | 1 | 0.995 | 1.00 (0.57, 1.75) |
| Intellect not extremely important | -0.162 | 1 | 0.397 | 0.85 (0.59, 1.24) |
| Aptitude not extremely important | 0.233 | 1 | 0.238 | 1.26 (0.86, 1.86) |
| Earnings not extremely important | -0.59 | 1 | 0.815 | 0.94 (0.58, 1.55) |
| Work-life balance not extremely important | 0.477 | 1 | 0.012 | 1.61 (1.11, 2.34) |
| Location not extremely important | -0.160 | 1 | 0.365 | 0.85 (0.60, 1.21) |
| Constant | -0.953 | 1 | 0.002 |  |

**Addtional file 1: Table S15: Logistic regression model predicting whether paediatrics is top three choice for Year 5 students**

|  | **Coeff** | **df** | **p-value** | **Odds ratio (95% CI)** |
| --- | --- | --- | --- | --- |
| Country of birth |  | 2 | 0.857 |  |
| Rest of UK (Scotland reference) | 0.079 | 1 | 0.647 | 1.08 (0.77, 1.52) |
| Other (Scotland reference) | -0.048 | 1 | 0.864 | 0.95 (0.55, 1.64) |
| Medical school |  | 3 | 0.010 |  |
| Dundee (Aberdeen reference) | -0.118 | 1 | 0.604 | 0.89 (0.57, 1.39) |
| Edinburgh (Aberdeen reference) | 0.524 | 1 | 0.016 | 1.69 (1.10, 2.58) |
| Glasgow (Aberdeen reference) | 0.377 | 1 | 0.092 | 1.46 (0.94, 2.26) |
| Male gender | -0.853 | 1 | <0.001 | 0.43 (0.30, 0.62) |
| Age (22 and over reference) | 0.107 | 1 | 0.657 | 1.11 (0.69, 1.79) |
| SEC (1&2 reference) | -0.102 | 1 | 0.650 | 0.90 (0.58, 1.40) |
| Non-white ethnicity | 0.019 | 1 | 0.944 | 1.02 (0.61, 1.71) |
| Intellect not extremely important | -0.33 | 1 | 0.853 | 0.97 (0.69, 1.37) |
| Aptitude not extremely important | 0.007 | 1 | 0.968 | 1.01 (0.70, 1.44) |
| Earnings not extremely important | 0.336 | 1 | 0.168 | 1.40 (0.87, 2.26) |
| Work-life balance not extremely important | -0.032 | 1 | 0.857 | 0.97 (0.68, 1.37) |
| Location not extremely important | 0.107 | 1 | 0.517 | 1.11 (0.81, 1.54) |
| Constant | -1.373 | 1 | <0.001 |  |

**Addtional file 1: Table S16: Logistic regression model predicting whether diagnostics is top three choice for Year 5 students**

|  | **Coeff** | **df** | **p-value** | **Odds ratio (95% CI)** |
| --- | --- | --- | --- | --- |
| Country of birth |  | 2 | 0.572 |  |
| Rest of UK (Scotland reference) | -0.192 | 1 | 0.515 | 0.83 (0.46, 1.47) |
| Other (Scotland reference) | 0.244 | 1 | 0.541 | 1.28 (0.58, 2.80) |
| Medical school |  | 3 | 0.017 |  |
| Dundee (Aberdeen reference) | -0.659 | 1 | 0.042 | 0.52 (0.27, 0.98) |
| Edinburgh (Aberdeen reference) | -1.109 | 1 | 0.004 | 0.33 (0.16, 0.70) |
| Glasgow (Aberdeen reference) | -0.625 | 1 | 0.068 | 0.54 (0.27, 1.05) |
| Male gender | 0.57 | 1 | 0.026 | 1.78 (1.07, 2.94) |
| Age (22 and over reference) | 0.263 | 1 | 0.439 | 1.30 (0.67, 2.53) |
| SEC (1&2 reference) | -0.055 | 1 | 0.877 | 0.95 (0.48, 1.89) |
| Non-white ethnicity | -0.167 | 1 | 0.685 | 0.85 (0.38, 1.89) |
| Intellect not extremely important | -0.622 | 1 | 0.035 | 0.54 (0.30, 0.96) |
| Aptitude not extremely important | -0.021 | 1 | 0.947 | 0.98 (0.53, 1.80) |
| Earnings not extremely important | -0.332 | 1 | 0.305 | 0.72 (0.38, 1.35) |
| Work-life balance not extremely important | -0.260 | 1 | 0.368 | 0.77 (0.44, 1.36) |
| Location not extremely important | -0.058 | 1 | 0.826 | 0.94 (0.56, 1.58) |
| Constant | -1.511 | 1 | <0.001 |  |
